# Adsorptive Removal and Adsorption Kinetics of Fluoroquinolone by Nano-Hydroxyapatite

**DOI:** 10.1371/journal.pone.0145025

**Published:** 2015-12-23

**Authors:** Yajun Chen, Tao Lan, Lunchao Duan, Fenghe Wang, Bin Zhao, Shengtian Zhang, Wei Wei

**Affiliations:** 1 Jiangsu Provincial Key Laboratory of Materials Cycling and Pollution Control, School of Geography Science, Nanjing Normal University, Nanjing, 210023, China; 2 Jiangsu Center for Collaborative Innovation in Geographical Information Resource Development and Application, Nanjing, 210023, China; 3 Nanjing Institute of Environmental Sciences, Ministry of Environmental Protection of China, Nanjing, 210042, PR China; Stem Cell Research Institute, BELGIUM

## Abstract

Various kinds of antibiotics, especially fluoroquinolone antibiotics (FQs) have been widely used for the therapy of infectious diseases in human and livestock. For their poorly absorbed by living organisms, large-scale misuse or abuse of FQs will foster drug resistance among pathogenic bacteria, as well as a variety of environmental problems when they were released in the environment. In this work, the adsorption properties of two FQs, namely norfloxacin (NOR) and ciprofloxacin (CIP), by nano-hydroxyapatite (n-HAP) were studied by batch adsorption experiments. The adsorption curves of FQs by n-HAP were simulated by Langmuir and Freundlich isotherms. The results shown that NOR and CIP can be adsorbed effectively by the adsorbent of n-HAP, and the adsorption capacity of FQs increase with increasing dosage of n-HAP. The optimum dosage of n-HAP for FQs removal was 20 g·L^-1^, in which the removal efficiencies is 51.6% and 47.3%, and an adsorption equilibrium time is 20 min. The maximum removal efficiency occurred when pH is 6 for both FQs. The adsorption isotherm of FQs fits well for both Langmuir and Freundlich equations. The adsorption of both FQs by n-HAP follows second-order kinetics.

## Introduction

Fluoroquinolones (FQs) such as norfloxacin (NOR) and ciprofloxacin (CIP) are a large class of antibiotics that are widely used in aquiculture, livestock husbandry, and human prescriptions [1–4]. However, FQs are often poorly absorbed, and are largely excreted in their pharmacologically active forms and metabolites after being applied to animals or humans [2, 5–7]. According to related reports, FQs are the most frequently detected antibiotics in wastewaters and surface waters, followed by sulfonamides, tetracyclines and macrolides [8, 9]. Relatively high concentrations (ng·L^-1^ to several μg·L^-1^) of FQs have been detected in domestic wastewater, municipal wastewaters, wastewater treatment plant (WWTP) effluents, surface waters and effluents from drug manufacturers all over the world [10–13]. Verlicchi et al. found the highest concentrations of FQs (over 100 μg·L^-1^) in hospital wastewater [1, 9]. Although typically present at vestigial levels, antibiotics may cause resistance in bacterial populations, which may make them ineffective in the treatment of several diseases in the near future [14–16]. Therefore, the presence of antibiotics in the environment has become an emerging concern.

Various methods for the removal of antibiotics have been applied to purify water, such as conventional techniques (biological processes, filtration, coagulation/flocculation and sedimentation) [17–21], advanced oxidation processes (AOPs) [22], adsorption [23], membrane processes [24], ozonation [25, 26], photochemical degradation [1, 27], and Fenton/photo-Fenton processes [28]. Among the above water-treatment techniques described, adsorption is generally preferred for the removal of FQs due to its relevant characteristics of high efficiency, easy handling, availability of different adsorbents, and cost effectiveness [29]. Clay minerals [30], natural zeolite [31] and carbon nanotubes had been studied for their adsorptive removal ability of FQs, among which actived carbon showed well performance [23]. However, the application of other adsorbent materials for FQs removal is also receiving considerable attention [32].

Nano-hydroxyapatite [Ca_10_(PO_4_)_6_(OH)_2_] (n-HAP) is a very well-known inorganic material in the biological and medical fields [33–36], and can be used as an adsorbent for the removal of heavy metals and dyes, due to its low water solubility, high stability under reducing and oxidizing conditions, high specific surface area, and good buffering properties [37, 38]. Application of hydroxyapatiteas drug carrier have been researched [39–42], Kumar synthesized HAP-ciprofloxacin powders by precipitation technique and investigated the adsorption and release process which may be an effective treatment of bacterial bone infections, and the adsorption was up to maximum in 24 hours while the release was slow and sustained for several weeks [41]; Yin et al. studied the HAP adsorption of histatins 1,3 and 5 and concluded that histatin 5 could be impaired by mineral adsorption [42]. However, its adsorption performance for antibiotics wastewater, especially for FQs, has not been well reported yet.

In this work, n-HAP was used as an adsorbent to study its adsorption capacity for NOR and CIP from aqueous solutions. The effects of adsorption time, n-HAP dosage and pH on its adsorption performance were determined. The adsorption curves were modeled according to the Langmuir and Freundlich isotherms. The adsorption kinetics and adsorption mechanism were estimated in terms of both the experimental and theoretical aspects.

## Materials and Methods

### Ethics statement

No specific permits were required for the described studies, and the work did not involve any endangered or protected species.

### Chemicals and materials

The pharmaceutical CIP was obtained from Guangdong Eashu Pharmaceutical Co., Ltd. NOR was purchased from Hangzhou Minsheng Pharmaceutical Co., Ltd. All the reagents were used without further purification. Other materials used included membrane filters (0.45 μm), Erlenmeyer flasks, drying oven (DHG-9240A, Shanghai Jing Hong Laboratory Instrument Co., Ltd), electronic balance (TP-214, Denver Instrument Co., Ltd), UV-vis spectrophotometer (UV2550, Shimadzu, Japan), X-ray diffraction (D/max 2500VL/PC, Rigaku, Japan), infrared spectrophotometer (Nexus 670, Nicolet, US), and transmission electron microscope (H-7650, Hitachi, Japan).

n-HAP was synthesized by a chemical precipitation method [43], which was considered to be an effective route to obtain HAP nanocrystals with lower crystallinity, smaller size and greater surface area. In order to characterize n-HAP used, X-ray diffraction (XRD), infrared spectrometry (IR), and transmission electron microscope (TEM) analysis were carried out. IR was used with KBr pellets in Nexus 670. TEM examination of the n-HAP samples and n-HAP after adsorption were carried out in a Hitachi Model H-7650 transmission electron microscope (TEM) operated at 80 kV. The TEM samples were prepared by depositing a few drops of the HAP powders ultrasonically dispersed in ethanol for 20 min on a carbon-coated copper grid.

### Batch adsorption experiment

The initial and residual concentrations of FQs in the aqueous samples were measured using a UV-vis spectrophotometer. The absorbance values of NOR and CIP were measured at wavelengths of 273 and 275 nm, respectively. All standard solutions were obtained by diluting the stock solutions with distilled water. The concentration of the NOR and CIP stock solution was 20 mg·L^-1^, and their initial pH of NOR and CIP were 7.24 and 6.59, respectively.

The calibration plot of absorbance values versus concentrations of NOR and CIP showed a linear variation within its experimental concentration range (0–25 mg·L^-1^), and the correlation coefficients (*R* ^2^) of both FQs were higher than 0.999, which indicated a suitably linear variation.

Various dosages of n-HAP (5, 25, 50, 250, 500 and 1000 mg) were added into 100 mL stoppered conical flask containing 50 mL test solution (20 mg·L^-1^ of NOR or CIP). Batch adsorption experiments were carried out on a temperature-controlled orbital shaker at 250 rpm and 25°C with 3 times. The residual FQs concentrations were analyzed at regular time intervals until reaching an approximately constant value. Samples were detected after filtration with a 0.45-μm disposable membrane. The removal efficiency *w* (%) and adsorption capacity *q* (mg·g^-1^) of NOR and CIP from the solution were calculated with the following equations.

$$w=\frac{c_{0}-c_{t}}{c_{0}}\times 100\%$$ (1)

$$q=\frac{\left(c_{0}-c_{t}\right)v}{m}$$ (2)

In which *c*_*0*_ is the initial antibiotic concentration (mg·L^-1^); *c*_*t*_ is the residual antibiotic concentration (mg·L^-1^); *v* is the volume of the solution (L); and *m* is the adsorbent dosage (g).

## Results and Discussion

### Characterization of n-HAP

**Fig 1:**
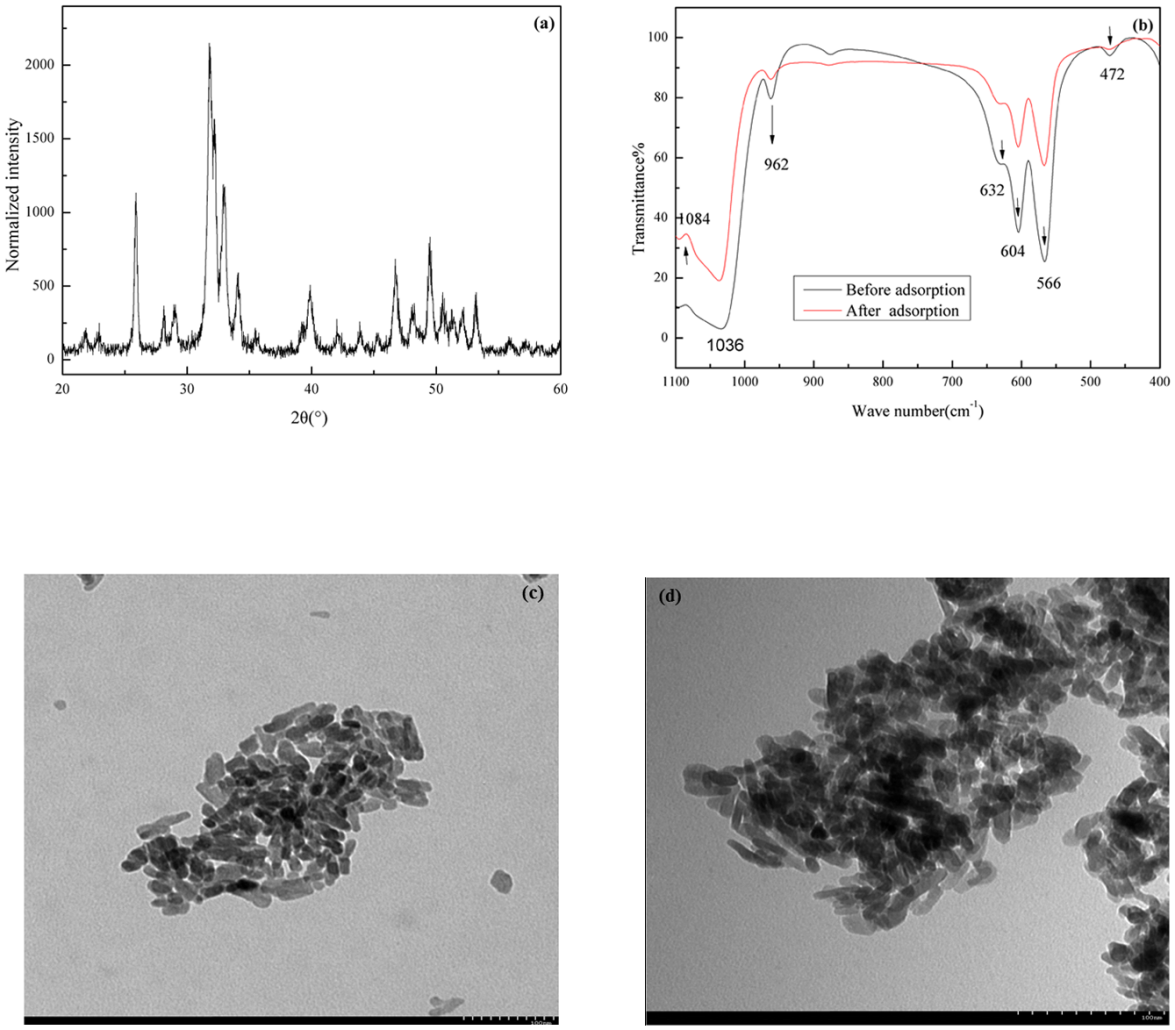
XRD, IR spectra and TEM image of n-HAP. (a) XRD. (b) IR of the n-HAP samples and n-HAP after adsorption. (c) TEM of n-HAP. (d) TEM of n-HAP after adsorption.

A typical XRD pattern of an n-HAP sample is shown in Fig 1(a).

By applying the Scherrer formula to the full width at half maximum of the diffraction peaks [44], we could calculate the average particle size of nano-crystalline HAP as 32.4 nm. The mineralogical identity and the crystallinity of n-HAP were confirmed.

The IR spectra of n-HAP before and after FQs adsorption in the wavelength range of 1100–400 cm^-1^ with KBr pellets in Nexus 670 are shown in Fig 1(b). All the characteristic bands of HAP that located at 1087, 1046, 962, 630, 601 and 472 cm^-1^ according reference of [45] can be seen in Fig 1(b). Compared with the original n-HAP, IR spectra of the solid residues after adsorption suggested that no other phases formed after it finished FQs absorption.

The TEM images of n-HAP are shown in Fig 1(c) and 1(d). As shown in Fig 1, the microscope of original n-HAP and the solid residues after adsorption have no changes.

### Effect of adsorption time on FQs adsorption

**Fig 2:**
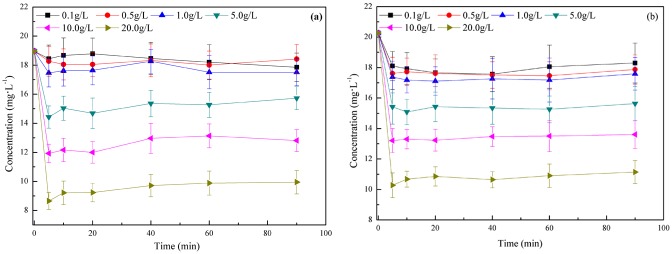
Effects of adsorbent dosage and adsorption time on the adsorption of FQs (25°C, NOR and CIP concentration were 20 mg/L, their pH of was 7.24 and 6.59, respectively). (a) For NOR. (b) For CIP.

Adsorbent dosage and adsorption time are important when studying the adsorption capacity at a given concentration of adsorbate [37]. The effects of adsorbent dosage and adsorption time on the residual concentration and removal efficiency of FQs are shown in Fig 2.

The effect of adsorption time was analyzed by varying time in the range of 5–90 min at 25°C temperature. As shown in Fig 2(a) and 2(c), the FQs concentration (NOR and CIP) decreased drastically in the first 5 min, then a slow decrease occurred until equilibrium was reached at 20 min. Generally, more time is needed to reach equilibrium; however, a very short time was required in this experiment. Having the small particle size of the adsorbent used, n-HAP can eliminate mass transfer limitations, and accelerate the adsorption process, previous studies have proved the adsorption efficiency is inversely proportional to the square of the adsorbent particle diameter [46, 47].

Taking the n-HAP dosage of 20 g·L^-1^ as an example, the removal efficiency of NOR from aqueous solution increased rapidly, and then reached 54.3% at 5 min. However, the removal efficiency was 49.3% for CIP. As shown in Fig 2, the removal efficiency decreased when the adsorption time increased. Changes in the removal curve were not significant after 20 min, which indicated that it was considered as the equilibrium time. Adsorption is a complex process, which comprised rapid adsorption, basic balance and perfect balance [48]. Namely, the adsorption of FQs (NOR and CIP) by n-HAP includes the processes of both adsorption and desorption. The fact that the desorption capacity is greater than the adsorption capacity is the main reason for the decrease in removal efficiency with increasing adsorption time. The aggregation of NOR around the n-HAP particles is another reason for this decrease. This is because aggregation may hinder the migration of adsorbate as the adsorption sites become filled up, and because resistance to the diffusion of FQs (NOR and CIP) molecules increases with increased adsorbent [49]. The reason for the adsorption efficiency that not constant after 3h is due to the experimental mistake.

### Effect of adsorbent dosage on FQs adsorption

The initial concentration of NOR was 20 mg·L^-1^ and the dosage of n-HAP varied from 0.1 to 1.0 g·L^-1^. In this concentration range, the dosage of n-HAP is apparently not enough to cause a decrease for NOR, because of limited surface area and reaction sites. Additionally, it was found that the adsorption capacity rise with increasing dosage of n-HAP. The residual concentration of NOR in solution decreased to 14.7 mg·L^-1^ at 5 g·L^-1^, 12.0 mg·L^-1^ at 10 g·L^-1^ and 9.2 mg·L^-1^ at 20 g·L^-1^, with removal efficiencies of 22.5%, 36.7% and 51.6%, respectively. This increase occurred because the adsorption capacity depends on the external surface area of the adsorbent, which increases with increasing of mass [37]. It can also contribute to the availability of more adsorption sites [50]. Additionally, a large dosage of n-HAP, with correspondingly large surface area and reaction sites, provides an important driving force to counteract the resistance to FQs mass transfer between the aqueous and solid phases [51]. Thus, a higher adsorbent dosage will increase the adsorption capacity for FQs.

For CIP, at an initial concentration of 20 mg·L^-1^, the removal efficiency of CIP varied between 9% and 13% when the dosages of n-HAP was from 0.1 to 1.0 g·L^-1^. The concentration of CIP in solution decreased to 15.1 mg·L^-1^ at 5 g·L^-1^, 13.3 mg·L^-1^ at 10 g·L^-1^ and 10.7 mg·L^-1^ at 20 g·L^-1^, with removal efficiencies of 25.5%, 34.3%, and 47.3%, respectively.

### Effect of pH

**Fig 3:**
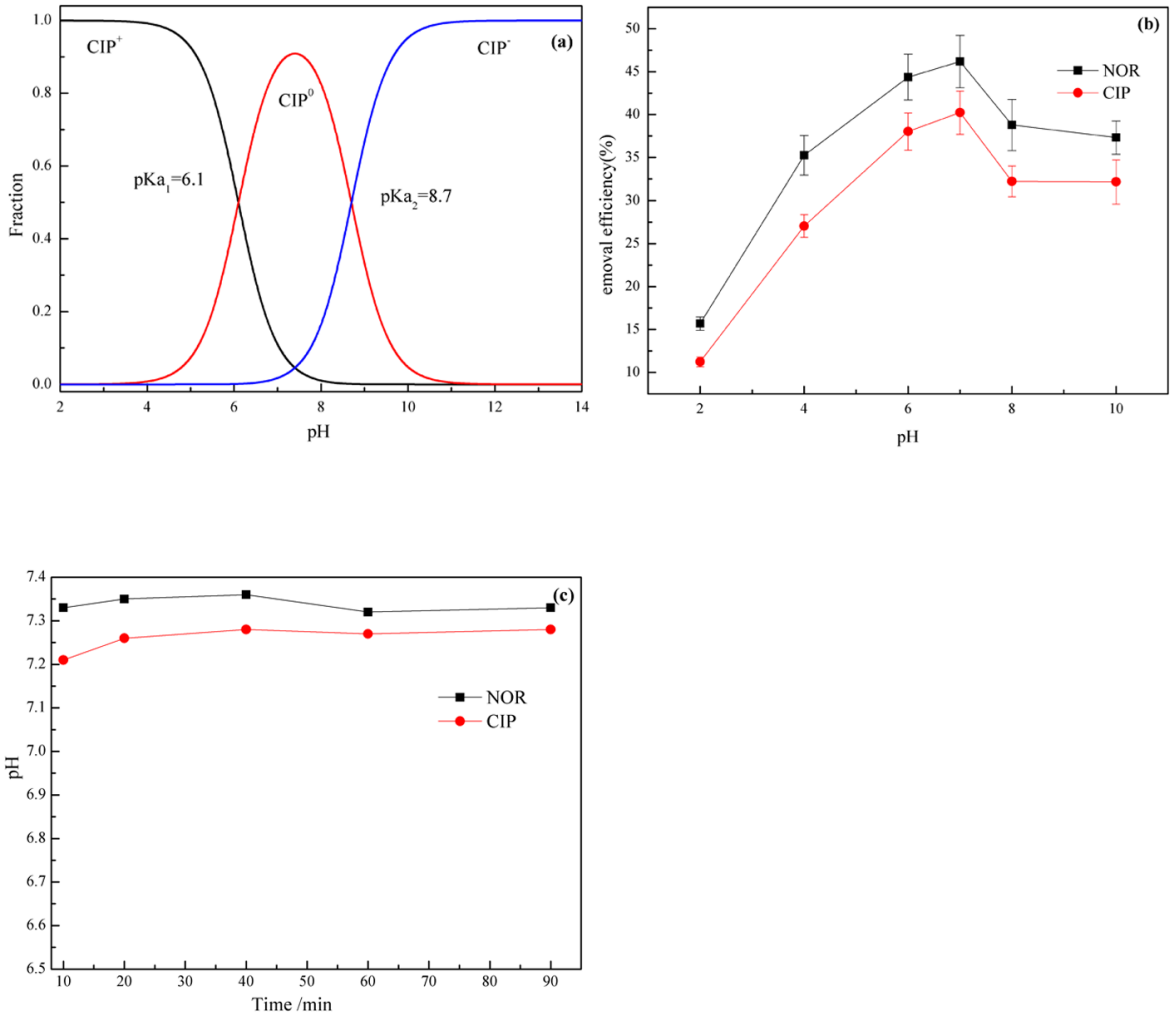
The effect of initial pH of FQs solution on FQs' removal (25°C, initial concentrations of FQs and HAP was 20mg/L and 500mg/L). (a) CIP distribution as a function of pH. (b) Effect of pH on FQs' removal efficiency. (c) Effect of time on pH.

pH is a significant factor for determining the removal of antibiotics in aqueous media. The effects of initial pH of FQs solution (from 2 to 10) on FQs' removal by 500 mg·L^-1^ n-HAP were studied. The results are shown in Fig 3.

As shown in Fig 3, the pH greatly influenced the adsorption of CIP onto n-HAP. The removal efficiency of FQs increased with the increasing of pH from 2 to 6. Then, the removal efficiency was reduced over the pH range from 6–10. In the acid solution, HAP will dissolve partly, which may decrease its adsorption efficiency. The dissolution of HAP is very low at pH 6 and pH 7, only 4 and 1%, respectively [52]. As shown in the Fig 3(c), adding n-HAP to the solution, the original pH did not change by a large margin. We affirmed that n-HAP did not dissolve when the pH of solution was near neutral. During this pH range, the pH effect on the removal efficiency of NOR was greater than that of CIP. The FQs removal efficiency in weakly acidic solution was higher (at pH = 6) than that in basic solution, which is consistent with the work of Van [37]. This phenomenon is attributed to the hydrophobic and electrostatic interactions between FQs in aqueous solution and the surface charge of n-HAP [53].

As shown in Fig 3(a), there were three kinds of CIP species in aqueous solution, namely, cationic CIP (CIP^+^), zwitterionic CIP (CIP^0^), anionic CIP (CIP^-^) due to its pKa values of 6.1 and 8.7 [54]. The distribution of the three kinds of CIP species can be calculated by Eqs (3)–(5):

$$\delta_{1}=\frac{{\left[H^{+}\right]}^{2}}{{\left[H^{+}\right]}^{2}+{\text{Ka}}_{1}*\left[H^{+}\right]+{\text{Ka}}_{1}*{\text{Ka}}_{2}}$$ (3)

$$\delta_{2}=\frac{{\text{Ka}}_{1}*\left[H^{+}\right]}{{\left[H^{+}\right]}^{2}+{\text{Ka}}_{1}*\left[H^{+}\right]+{\text{Ka}}_{1}*{\text{Ka}}_{2}}$$ (4)

$$\delta_{3}=\frac{{\text{Ka}}_{1}*{\text{Ka}}_{2}}{{\left[H^{+}\right]}^{2}+{\text{Ka}}_{1}*\left[H^{+}\right]+{\text{Ka}}_{1}*{\text{Ka}}_{2}}$$ (5)

Taking CIP as an example, pH has an important effect on the solubility of CIP due to its speciation. It can be seen that zwitterionic CIP has the highest hydrophobicity among the three ionic species, because of the lowest solubility at approximately pH 7 [55, 56]. The increased sorption of CIP can be attributed to the higher hydrophobicity of zwitterionic at approximately pH 7, generally considered to be an important factor for driving organic chemicals sorption [23].

Electrostatic interactions are considered to play an important role in the absorption of ionic compounds and have been successfully used to interpret the sorption of natural organic matter (NOM), phenolic chemicals and antibiotics [57, 58]. As shown in Fig 3(a), cationic CIP is dominant at pH < 6.1, while the dominant species is anionic at pH > 8.7. The surface of n-HAP is positively charged at pH<6.86 and negatively charged at pH>6.86 [59]. In this study, electrostatic repulsion occurs between CIP molecules and n-HAP surfaces at pH <6.1 and pH > 8.7, because the particles are both positively or negatively charged. The electrostatic repulsion reduces with an increase of pH up to 6.0, and increases in the pH range of 6–10. Over the pH range of 6.1–8.7, the electrostatic interaction may be weakened when hydrophobic interaction is enhanced and dominant due to its higher hydrophobicity of zwitterionic CIP.

### Adsorption isotherms

The adsorption isotherm is an important method that can be used to illustrate the adsorption properties between adsorbent and adsorbate, and is also an important theoretical basis for the practical application of adsorption, which is characterized by certain constants whose values express the surface properties and affinity of the sorbent sorption equilibrium [60]. The classic equations of Langmuir and Freundlich were used to fit the experimental equilibrium adsorption data using Eqs (6) and (7), respectively [61, 62]. The former equation assumes that adsorbate is only adsorbed on the adsorption sites of the adsorbent surface and is used to describe adsorption in a surface monolayer, in the form of specific adsorption. The latter equation is an empirical description of the adsorption equilibrium of a single component [63].

$$\frac{C_{e}}{Q_{e}}=\frac{1}{Q_{m}*K_{l}}+\frac{C_{e}}{Q_{m}}$$ (6)

$$lnQ_{e}=lnK_{f}+\frac{1}{n}*lnC_{e}$$ (7)

In which *C*_*e*_ (mg·L^-1^) and *Q*_*e*_ (mg·g^-1^) are the equilibrium concentrations of FQs in the liquid and adsorbent, respectively; *Q*_*m*_ (mg·g^-1^) is the maximum adsorption capacity according to the Langmuir model; *K*_*l*_ is a Langmuir constant associated with the adsorption intensity; *K*_*f*_ is a constant associated with the adsorption capacity for the Freundlich model; and *n* is concentration index associated with the adsorption intensity for the Freundlich model. Generally, adsorption occurs readily when the value of *n* is between 1 and 10.

**Fig 4:**
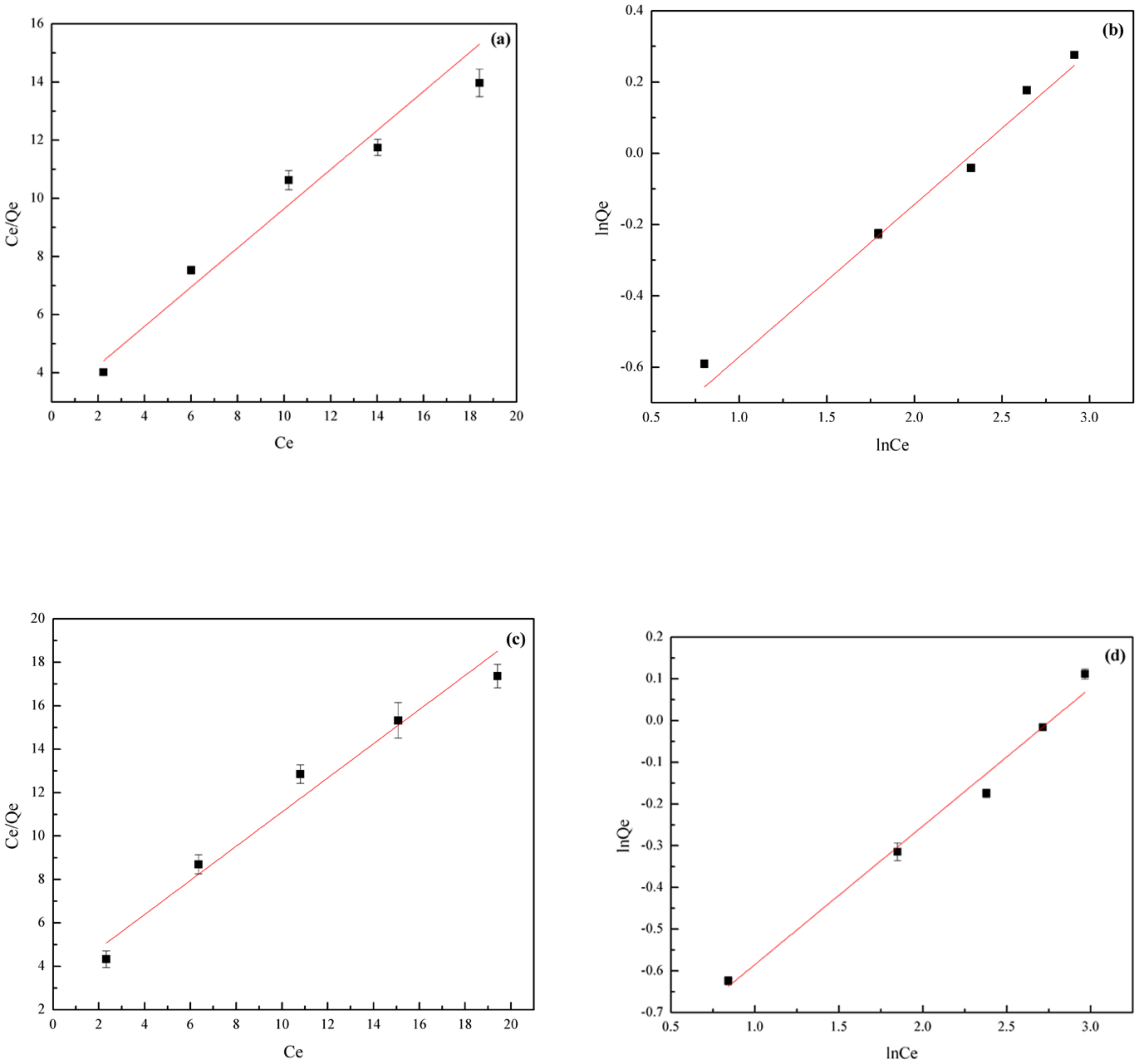
The adsorption isotherms of FQs on n-HAP. (a) Langmuir adsorption curve of NOR. (b) Freundlich adsorption curve of NOR. (c) Langmuir adsorption curve of CIP. (d) Freundlich adsorption curve of CIP.

On the basis of Eqs (6) and (7), the curves of the adsorption isotherms were obtained by calculating linear fits to the processed data in Fig 4.

The intercept and slope of each straight line were used to obtain the FQs isotherm parameters and were listed in Table 1.

**Table 1:** The adsorption isotherms parameters of NOR and CIP by n-HAP.

Isotherm equation	Parameter	NOR	CIP
**Langmuir**	*Q*_*m*_(mg·g^-1^)	1.4862	1.2715
**Langmuir**	*K*_*l*_	0.2308	0.02365
**Langmuir**	R^2^	0.94781	0.95633
**Freundlich**	*K*_*f*_	0.3684	0.3998
**Freundlich**	*1/n*	0.42761	0.33187
**Freundlich**	R^2^	0.96552	0.98792

The values of the Freundlich exponent (*n*) between 1 and 10 represent a favorable adsorption [64]. The values of *n*, which reflect the intensity of adsorption, also reflected the same trend. As shown in Table 1, the *n* values obtained for the adsorption process represented a beneficial adsorption [65]. Furthermore, the 1/*n* of NOR (0.42761) is more than that of CIP (0.33187), what made n-HAP having a preferential sorption for NOR. The adsorption capacity of FQs followed the order of NOR (1.4862 mg·g^-1^) > CIP (1.2715 mg·g^-1^), which demonstrated that n-HAP had a higher adsorption capacity for NOR compared with CIP. The Langmuir correlation coefficients R^2^ were determined to be 0.94781 and 0.95633 for NOR and CIP, respectively; while they were respectively 0.96552 and 0.98792 for the Freundlich equation. The high correlation coefficients indicated that the FQs adsorption isotherms can be described well by both Langmuir and Freundlich equations.

### Adsorption kinetics

The adsorption behavior of an adsorbent on an adsorbate can be studied by using adsorption kinetics. The adsorption efficiency is the change in adsorption per unit time and is the key parameter used to describe absorption efficiency [66]. First-order and second-order kinetic models expressed by Eqs (8) and (9) [67] were fitted to the experimental data. The interrelation between adsorption capacity and contact time was studied to clearly confirm the factors influencing the adsorption process.

$$ln\left(Q_{e}-Q_{t}\right)=lnQ_{e}-K_{1}*t$$ (8)

$$\frac{t}{Q_{t}}=\frac{1}{K_{2}*Q_{e}{}^{2}}+\frac{t}{{\text{Q}}_{e}}$$ (9)

Where *Q*_*t*_ (mg·g^-1^) is the concentration of FQs in the solid phase at any time; *Q*_*e*_ (mg·g^-1^) is the equilibrium concentration of FQs in the adsorbent; and *t* (min) is the adsorption time. The value of ln(*Q*_*e*_-*Q*_*t*_) or *t*/*Q*_*t*_ was linearly correlated with *t*. The plot of ln(*Q*_*e*_-*Q*_*t*_) or *t*/*Q*_*t*_ versus *t* should give a linear relationship, from which *K*_1_ (min^-1^) and *Q*_*e*_, or *Q*_*e*_ and *K*_2_ (g·mg^-1^·min^-1^), can be determined from the slope and intercept of the plot, respectively. *K*_1_ and *K*_2_ are the rate constants for the first-order and second-order models, respectively.

**Fig 5:**
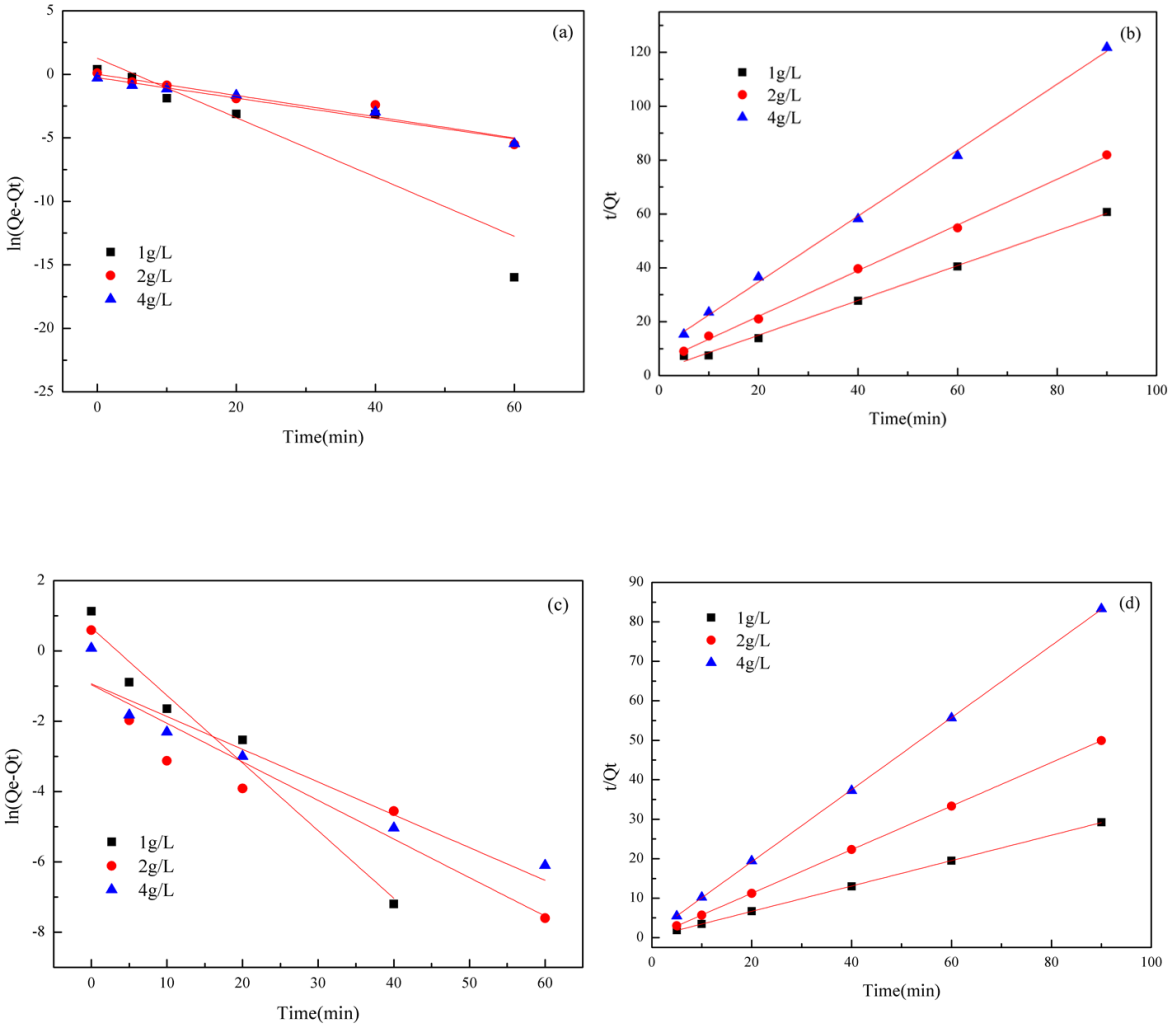
The adsorption kinetics plots of FQs on n-HAP. (a) First-order kinetics curve of NOR. (b) Second-order kinetics curve of NOR. (c) First-order kinetics curve of CIP. (d) Second-order kinetics curve of CIP.

The adsorption kinetics curves were calculated in Fig 5, with the contact time (*t*) as the abscissa (X), and ln(*Q*_*e*_-*Q*_*t*_) and *t*/*Q*_*t*_ as the vertical axis (Y), respectively. The adsorption kinetics of FQs by n-HAP materials (1 g·L^-1^, 2 g·L^-1^ and 4g·L^-1^) was evaluated.

In order to quantify the applicability of the models, the experimental *Qe* (*Q*_*e*_,_*exp*_) and calculated *Qe* (*Q*_*e*_,_*cal*_) values and other parameters calculated from the kinetic models were listed in Table 2. As shown in Table 2, all the R^2^ values obtained in pseudo-first-order kinetic model were relatively small, which implied that pseudo-first-order kinetic model did not adequately fit the experimental data well. The *Q*_*e*_,_*exp*_ and the *Q*_*e*_,_*cal*_ values from pseudo-second-order kinetic model were similar to each other, and their correlation coefficients of R^2^ were more than 0.99. Therefore, it can be concluded that the adsorption process of FQs onto n-HAP followed the pseudo-second-order kinetic model, and the adsorption mechanism might be chemisorption involving valence forces at some specific sorption sites [67, 68].

**Table 2:** The Adsorption Kinetics Parameters of NOR and CIP by n-HAP.

FQs	Contents	*Q*_*e*_,_*exp*_	First-order kinetics			Second-order kinetics		
			*Q*_*e*_,_*cal*_	*K*_*1*_	R^2^	*Q*_*e*_,_*cal*_	*K*_*2*_	R^2^
**NOR**	1 g·L^-1^	1.4822	3.5261	0.2336	0.7514	1.5477	0.2030	0.9963
**NOR**	2 g·L^-1^	1.0985	1.0111	0.0837	0.9236	1.1791	0.1425	0.9986
**NOR**	4 g·L^-1^	0.7390	0.7673	0.0804	0.965	0.8172	0.1459	0.9981
**CIP**	1 g·L^-1^	3.0810	1.9284	0.1923	0.9592	3.1113	0.4577	0.9999
**CIP**	2 g·L^-1^	1.8048	0.3820	0.1097	0.8337	1.8109	1.5550	0.9999
**CIP**	4 g·L^-1^	1.0783	0.3929	0.0931	0.9124	1.0948	0.8779	0.9999

## Conclusion

n-HAP is a useful adsorbent for removing FQs from aqueous solution. The adsorption capacity of NOR and CIP increased with dosage increasing of n-HAP. The maximum removal efficiencies were 54.3% and 47.3% at 20 g·L^-1^ n-HAP for NOR and CIP, respectively, with an equilibrium time of 20 minute. pH played an important role in the removal of FQs. The removal efficiency in weak acidic solution (pH = 6–8) was higher than that in basic solutions. It was found that the adsorption isotherms of n-HAP could be described well by both Langmuir and Freundlich equations. However, the Freundlich isotherm fitted better than the Langmuir isotherm for NOR and CIP adsorption. The adsorption kinetics of NOR and CIP on n-HAP followed the second-order model well.
